# Hierarchically Structured Porous Piezoelectric Polymer Nanofibers for Energy Harvesting

**DOI:** 10.1002/advs.202000517

**Published:** 2020-06-03

**Authors:** Mohammad Mahdi Abolhasani, Minoo Naebe, Morteza Hassanpour Amiri, Kamyar Shirvanimoghaddam, Saleem Anwar, Jasper J. Michels, Kamal Asadi

**Affiliations:** ^1^ Max‐Planck Institute for Polymer Research Ackermannweg 10 Mainz 55128 Germany; ^2^ Chemical Engineering Department University of Kashan Kashan 8731753153 Iran; ^3^ Carbon Nexus Institute for Frontier Materials Deakin University Geelong 3217 Australia; ^4^ School of Chemical & Materials Engineering National University of Sciences & Technology Sector H‐12 Islamabad Pakistan

**Keywords:** finite element simulations, nanogenerators, phase diagrams, piezoelectric nanogenerators, porous nanofibers

## Abstract

Hierarchically porous piezoelectric polymer nanofibers are prepared through precise control over the thermodynamics and kinetics of liquid–liquid phase separation of nonsolvent (water) in poly(vinylidene fluoride‐trifluoroethylene) (P(VDF‐TrFE)) solution. Hierarchy is achieved by fabricating fibers with pores only on the surface of the fiber, or pores only inside the fiber with a closed surface, or pores that are homogeneously distributed in both the volume and surface of the nanofiber. For the fabrication of hierarchically porous nanofibers, guidelines are formulated. A detailed experimental and simulation study of the influence of different porosities on the electrical output of piezoelectric nanogenerators is presented. It is shown that bulk porosity significantly increases the power output of the comprising nanogenerator, whereas surface porosity deteriorates electrical performance. Finite element method simulations attribute the better performance to increased volumetric strain in bulk porous nanofibers.

## Graphical abstract

This work links the thermodynamics of the polymer/solvent/nonsolvent ternary system, to the hierarchically engineered porous structure in polymer nanofibers. The model is experimentally substantiated with piezoelectric polymer fibers, where it is shown that due to the porosity, the electrical output of comprising piezoelectric nanogenerators is significantly improved, which, though finite element simulations, is attributed to the increased volumetric strain in bulk porous nanofibers.

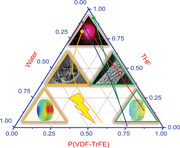

## 1 Introduction

Porous polymer structures due to their large specific surface area, light weight, and high adsorption capacity,^[^ ^1^ ^]^ are envisioned for variety of applications for instance in biomedicine,^[^ ^2^ ^]^ CO_2_ capture,^[^ ^3^ ^]^ filtration,^[^ ^4^ ^]^ and vibrational piezoelectric energy harvesting.^[^ ^5^, ^6^ ^]^ For energy harvesting it has been shown that the presence of pores in the polymer structures is beneficial as it leads to enhanced power output of the generator. The main challenge is however designing a robust process for the polymers that enables controlling of the (amount of) porosity and the position of the pores in the final structure, i.e., surface or bulk porosity.

Careful design of porous polymeric structures is a daunting task. An experimentally well‐established route to fabricate porous polymer films for membranes applications is to exploit thermodynamics of polymer/solvent/nonsolvent solutions and liquid–liquid phase separation,^[^ ^7^, ^8^ ^]^ which require establishing the ternary phase diagram of the system and a solid understanding of the kinetic of the phase separation process. The ternary phase diagram consists of three distinct regions; 1) the miscibility region where the solution is stable and a continuous compact layer upon casting is formed. 2) and 3) the immiscibility regions, which are divided into two distinct sections, i.e., metastable and unstable areas. Inside the metastable area, the thermodynamically unstable polymer solution is separated via a nucleation and growth mechanism (NG), while the spinodal decomposition (SD) is the mechanism dominating the phase separation process inside the unstable area. The NG phase separation leads to isolated pores, whereas the SD phase separation yields an interconnected network of pores.^[^ ^9^ ^]^ Since the polymer membranes are typically tens of micrometer thick, presence of pores of tens of nanometer to several micrometer does not compromise the structural integrity.

Engineering porosity in nanostructured assemblies, such as polymer nanofibers is still in its infancy. Polymer nanofibers are typically prepared by electrospinning and have a diameter that is only several hundred nanometers. Hence, presence of large pores adversely affects the integrity of single fibers. Creating porous nanofibers requires a careful study of the polymer/solvent/nonsolvent ternary phase diagram. Due to the large surface to volume ratio in nanofibers, solvent evaporation is much faster than in films. Therefore, the thermodynamics of phase separation during electrospinning and development of the porous nanostructure within electrospun fibers will be dominated by solvent volatility. Control over porosity can be achieved through careful selection of solvent and composition of the polymer/solvent/nonsolvent solution. To the best of our knowledge, there is a snippet of information as how to hierarchically engineer the porosity in a nanofiber such that pores can be positioned within the nanofiber or on the surface.^[^ ^10^ ^]^ Achieving this level of directing porosity requires a good control over the thermodynamics and kinetics of phase separation.

Porous piezoelectric structures can be used in variety of applications such as vibrational energy harvesters,^[^ ^11^ ^]^ where the piezoelectric material harvests waste mechanical energy from various sources such as walking, sonic wave, etc.^[^ ^5^, ^11^, ^12^ ^]^ The best performing piezoelectric polymer to date is poly(vinylidene difluoride), PVDF, and its random copolymers with trifluoroethylene, P(VDF‐TrFE), which are widely used as sensors and memories.^[^ ^13^ ^]^ The piezoelectric activity in PVDF strongly depends on crystalline structure, as the polymer can crystalize in five different polymorphs, namely *α*, *β*, *γ*, *δ*, and *ε*, of which the most favored and thermodynamically stable one upon melt or solution processing is the nonpolar, hence non‐piezoelectric, *α*‐phase.^[^ ^14^ ^]^ The commonly used piezoelectric *β*‐phase PVDF is made (usually in a combination with the *α*‐phase) by adding nanofillers,^[^ ^15^ ^]^ mechanical stretching,^[^ ^14^ ^]^ or processing in spatially confined geometries.^[^ ^16^ ^]^ In sharp contrast, the random copolymer P(VDF‐TrFE) advantageously crystallizes solely into the piezoelectric *β*‐phase irrespective of the processing conditions. Due to the absence of non‐polar polymorph at room temperature, and that the polar form shows ferroelectric properties, P(VDF‐TrFE) has been extensively exploited for non‐volatile memory application.^[^ ^27^ ^]^ Therefore, because of the presence of only one piezoelectric/ferroelectric crystallographic phase,^[^ ^28^ ^]^ P(VDF‐TrFE) is an ideal model system for the study of the effect of porosity on the piezoelectric properties of the nanofibers. However, since P(VDF‐TrFE) is usually processed from hygroscopic solvents, creating porous nanofibers with controllable and hierarchical porosity is by no means trivial.

Here we present selective localization of the pores on the surface or inside the bulk of P(VDF‐TrFE) piezoelectric nanofibers through combination of solvent selection and deliberate addition of a certain amount of water (non‐solvent) to the comprising solutions. Since the solvents, i.e., tetrahydrofuran (THF) and dimethylformamide (DMF) are both hygroscopic, purposeful addition of water in metastable and unstable areas of phase diagram to control the amount of porosity is not trivial and requires good understanding of the kinetic of phase separation. To do so, P(VDF‐TrFE)/THF (and DMF)/water ternary phase diagrams are calculated first and experimentally verified, from which mass transfer trajectories have been calculated by taking solvent evaporation, as well as water evaporation and condensation. Finally, we have exploited the porous piezoelectric nanofibers for energy harvesting from mechanical vibrations. We show that while surface porosity deteriorates the performance of nanogenerators bulk porosity significantly increase the power output of the porous fibers by up to 280 times compared to reference P(VDF‐TrFE) fibers. Finite‐Element simulation method is performed to pinpoint the origin of the enhanced power output of the nanogenerators. The presented method can be adopted to provide guideline for designing various nanostructures suited for applications other than energy harvesters, for example in electronic textiles, triboelectric nanogenerators, filtration membranes, batteries and catalysis.

## 2 Experimental Section

### 2.1 Materials

Poly(vinylidenefluoride‐*co*‐trifluoroethylene) P(VDF‐TrFE), (VDF/TrFE = 70/30) was purchased from Arkema and tetrahydrofuran (THF), dimethylformamide (DMF) and ethylene glycol (EG) were supplied from Sigma Aldrich, Inc.

### 2.2 Methods

#### 2.2.1 Solution Properties and Electrospinning Procedure

15 wt% polymer solutions stirred overnight were used for electrospinning at room temperature. Electrospinning experiments were conducted at voltage of 20 kV while the tip to collector distance was adjusted at 17 cm and feeding rate was 1 mL h^−1^. The electrospinning lasted for 5 h to produce fiber mats with a thickness of 50 µm. Two series of dopes with DMF and THF were prepared. Dopes DMF1 and DMF2 contain DMF as solvent and 0.0 and 2 wt% of water as non‐solvent. Dopes THF1‐THF4 have THF as solvent and 0.0, 5.1, 10.2, and 12.7 wt% water as non‐solvent.

#### 2.2.2 Cloud Point Measurements

P(VDF‐TrFE)/DMF and P(VDF‐TrFE)/THF solutions of different concentrations from 1 to 20 wt%, were prepared for determining the cloud points. Afterward, water was added slowly to the P(VDF‐TrFE) solution till the initial solution turned turbid.

#### 2.2.3 Fiber Characterization

Surface and cross‐section morphology of electrospun fibers were evaluated using scanning electron microscopy (SEM, Zeiss SupraTM 55VP). To prepare samples for cross‐sectional SEM imaging of the nanofibers, the mats were immersed in poly(vinylalcohol) water solution (20 wt% ) for 1 min and subsequently dried in vacuum oven overnight. The resulted P(VDF‐TrFE)/PVA composites was immersed in liquid nitrogen and then broken for cross‐sectional imaging.

#### 2.2.4 Porosimetry

Thermoporometry was used to evaluate the porosity of electrospun P(VDF‐TrFE) fibers. To this end, the electrospun mat with known dry weight was wetted by the probe liquid, ethylene glycol, by immersion into the ethylene glycol for 48 h. The wetted mat was weighed and used for thermoporometry. For this purpose, a certain mass (around 5–10 mg) of wetted sample was put into a hermetic pan and placed in differential scanning calorimetry (DSC, TA, Q200) instrument under the nitrogen atmosphere. Samples were immediately quenched to −60 °C, far below the equilibrium freezing temperature of EG, and maintained at this temperature for 30 min. The quenched sample was then slowly heated to 0 °C with heating rate of 0.5 °C min^−1^ to ensure the instalment of thermodynamic equilibrium. Having the mass of the dry and wetted mat, it is possible to calculate the mass of ethylene glycol required for further calculations as explained in the Supporting Information.

#### 2.2.5 FTIR

Fourier transform infrared spectroscopy (FTIR) was done by 64 scans between 600 and 2000 cm^−1^ with a resolution of 4 cm^−1^ using Bruker 70.

#### 2.2.6 Nanogenerators

To fabricate the nanogenerator a 2 × 2 cm^2^ piece of the P(VDF‐TrFE) mats was sandwiched between two Al foils as electrodes. The generators were mechanically impacted at a frequency of 1 Hz and a pressure of 0.2 MPa using a home‐built set‐up. To investigate effect of poling on the performance, the nanofiber mats were poled after electrospinning, using a high‐voltage Radiant Multiferroic tester. However, no difference was observed between the piezoelectric voltage of the poled and unpoled samples, which demonstrate that the electropsun fibers are self‐poled,^[^ ^18^, ^19^, ^29^ ^]^ in accordance with previous reports. The self‐poling of P(VDF‐TrFE) nanofibers is due to the mechanical stretching of the nanofibers under the electric fields during the electrospinning process.

## 3 Results and Discussion

### 3.1 Ternary Phase Diagrams

The hierarchical porosity within the electrospun mats is precisely designed by using the thermodynamics and kinetics of liquid‐liquid phase separation in the ternary mixture of P(VDF‐TrFE), solvent (i.e., THF or DMF) and water (nonsolvent). In general, upon evaporation of the solvent the composition enters a miscibility gap,^[^ ^8^ ^]^ where the strongly repulsive interaction between the polymer and the non‐solvent drives demixing in a polymer‐rich and a polymer‐devoid phase. We expect this demixed structure to be readily “frozen in” upon gelling due to P(VDF‐TrFE) crystallite formation. As the total volume and spatial distribution of the water determines the porosity in the dry specimen, structure formation can be directed toward producing a desired morphology upon the addition of a controlled quantity of water to the initial polymer‐solvent mixture.^[^ ^5^ ^]^ However, as we have shown before, in case of a hygroscopic solvent, one has to consider the possibility that ambient water vapor may condense into the drying solution thereby altering the water content during processing. Alternatively, in dry conditions initially added water may evaporate from the solution before demixing takes place.

**Figure 1:**
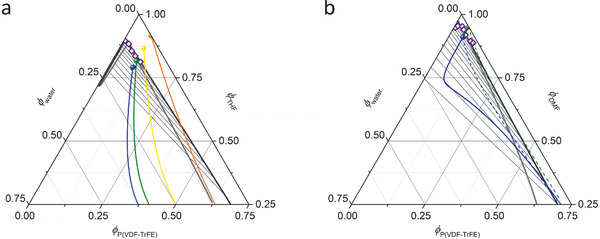
Calculated ternary phase diagrams for a) P(VDF‐TrFE)/THF/water and b) P(VDF‐TrFE)/DMF/water. The binodal curves (dark gray) have been fitted against experimentally determined cloud point compositions (purple diamonds). The spinodal curves are indicated in light gray and tie‐lines in black. The colored dots in diagram (a) indicate the initial compositions of dopes THF1 (orange), THF2 (yellow), THF3 (green), and THF4 (blue). The colored dots in diagram (b) indicate the initial composition of dopes DMF1 (green) and DMF2 (blue). The colored curves in diagrams (a,b) (green and blue overlapping) represent mean composition trajectories calculated for processing under conditions with elevated humidity; the dashed curves in diagram (b) represent the same calculation though assuming a higher effective mass transfer coefficient for DMF than water and a low effective humidity.

Our first step in achieving porous materials based on this phase inversion technique, is hence to obtain an estimate of the phase behavior of the P(VDF‐TrFE)/solvent/water mixtures by calculating ternary phase diagrams that fit experimental cloud point data (see the Experimental Section and Section S1 in the Supporting Information for the details of the calculations). The resulting ternary phase diagrams, as well as the cloud point compositions (purple diamonds), for P(VDF‐TrFE)/THF/water and P(VDF‐TrFE)/DMF/water are given in **Figure** **1**a,b, respectively. The calculated binodal curve (dark gray) with a good accuracy fits the experimentally determined cloud point data. The phase diagrams show how the binodal curve divides composition space in a single phase and a two‐phase region for both solvents. The thin black lines are tie‐lines that connect the coexisting phases in composition space. The spinodal curve (light grey) divides the coexistence region in a metastable and unstable region. In the metastable region, the solution phase separates via NG, whereas SD is the mechanism dominating the phase separation in the unstable region. The colored dots in the phase diagrams indicate the initial compositions of the THF (Figure 1a) and DMF (Figure 1b) based polymer solutions (“dopes”) that we have used for electrospinning the fiber mats. The colored lines indicate calculated trajectories of mean composition associated with the drying of the solutions. In the following, we discuss the relation between the way these trajectories traverse the phase diagram and the morphology of the dry fibers.

Comparison of Figure 1a,b reveals that the miscibility gap for the THF‐based compositions is smaller than for DMF. This means that in the latter a stronger driving force for phase separation exists, already at low water content. The significant tilt in the tie‐lines in both diagrams shows that the polymer‐rich phase is highly concentrated and the water content in the coexisting phases is comparable for both solutions. The large miscibility gap and steep tie‐lines are in good agreement with previous calculations considering P(VDF‐TrFE)/solvent/water mixtures, based on Flory‐Huggins theory.^[^ ^5^, ^20^ ^]^

### 3.2 Nanofiber Morphology

**Figure 2:**
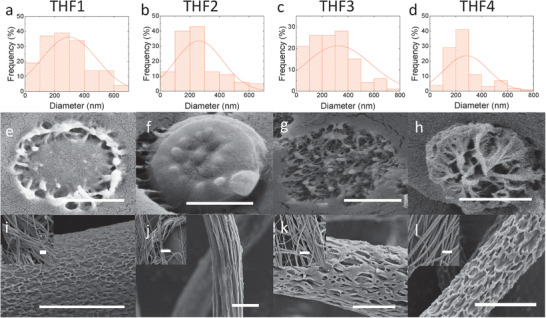
a–d) the nanofiber diameter distribution histograms of THF1‐THF4. e–h) SEM images of the cross‐sectional view of a single fiber. i–l) SEM images from surface of a single fiber. The insets represent low magnification images of the nanofibers. The scale bars are 200, 500, and 500 nm for the cross‐section, surface and insets images, respectively.

**Figure 3:**
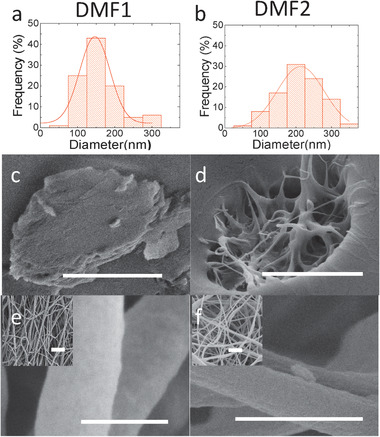
a,b) nanofiber diameter distribution histograms of DMF1 and DMF2. c,d) SEM images of the cross‐sectional view of a single fiber. e,f) SEM images from surface of a single fiber. The insets represent low magnification images of the nanofibers. The scale bars are 200, 500, and 500 nm for the cross‐section, surface and insets images, respectively.

**Figures** **2** and **3** show SEM images (surface and cross‐sectional) of P(VDF‐TrFE) nanofibers electrospun from dopes THF1‐4 and DMF1‐2 and their corresponding diameter distribution, respectively. The insets are low‐magnification top‐view SEM images of the resulting “non‐woven” nanofiber mats. The dopes respectively contained 0, 5.1, 10.2, and 12.7 wt% (THF‐1 to 4 dopes), and 0.0 and 2.0 wt% (DMF1‐2 dopes) of water (see the Experimental Section). The cross sectional images (middle panels) demonstrate that the bulk porosity inside the nanofibers is related to the amount of added water. Samples without added water, i.e., THF1 and DMF1, clearly show a solid core structure, whereas for DMF2, THF3, and THF4 L‐L phase separation results in a porous morphology. A notable exception is THF2, for which a solid core is obtained despite the initial presence of 5.1% of water. All bulk porosities have been quantified by means of thermoporometry (see the Experimental Section) and are listed in **Table** **1**. All fibers electrospun from the THF‐based dopes display a porous surface structure (see Figure 2). In contrast, the fibers electrospun from DMF‐dopes (Figure 3) have a smooth surface, i.e., in agreement with previous reports.^[^ ^5^ ^]^

**Table 1:** Average diameter and estimated pores volume fraction of P(VDF‐TrFE) electrospun fibers

Electrospinning Dope	THF1	THF2	THF3	THF4	DMF1^a)^	DMF2^a)^
Diameter^[^ ^21^ ^]^	299 ± 185	262 ± 162	322 ± 255	281 ± 159	137 ± 37	181 ± 55
Pore volume Fraction [%]	0	0	31	45	0	45
Crystallinity [%]	23	23	24	24	31	40

a) Adopted from reference.^[^ ^5^ ^]^

The bulk porosity is driven by phase separation induced by the initial water content and/or water ingress into the drying fiber upon leaving the nozzle and/or after having landed on the substrate. In contrast, the surface‐porosity in the THF‐processed fibers is likely formed in a “breath‐figure”‐type process wherein a steep decrease in the local temperature due to heat‐loss upon fast evaporation causes water nanodroplets to condense and precipitate onto the drying fiber.^[^ ^22^ ^]^ The fact that no breath figure structures are observed in fibers processed from DMF, is consistent with the much slower evaporation rate: the vapor pressure of DMF is roughly a factor 40 lower than that of THF.^[^ ^23^ ^]^

We note that for the same reason of having a very low vapor pressure, the rate of evaporation of DMF is also lower than that of water. Consequently, the difference in behavior of the two DMF dopes is not straightforwardly explained by a previously proposed evaporation model based on a partial pressure deficit,^[^ ^5^, ^20^ ^]^ and assuming the liquid to be in thermal equilibrium with the region in the vapor phase just above it. Taking the ambient partial pressure of DMF and water to be, respectively 0 and 1167 Pa (i.e., ≈50% RH at ≈20 °C), the calculated composition trajectories (green and blue solid lines in Figure 1b) overlap, suggesting that the mean composition is at any time dominated by the ambient humidity. Only by assuming the effective transfer rate of DMF to be higher than that of water (e.g., by a factor ≈10), together with a ≈5 × lower effective ambient partial water pressure, the model produces a scenario wherein the composition of the dry dope (DMF1) does not significantly overlap with the miscibility gap, whereas that of the wet dope (DMF2) does (dashed green and blue lines). A possible explanation for this discrepancy is the formation of an initial skin layer at the periphery of the drying fiber, that allows still allows for DMF to evaporate but suppresses the ingress of ambient water.

In contrast to the DMF dopes, the model more straightforwardly explains the (qualitative) difference in behavior between the THF solutions (see Figures 1a and 2). Since the evaporation of THF is fast in comparison to the evaporation or ingress of water, the direction of the mean composition trajectories is dominated by the initial water content. The orange trajectory, i.e., representing the fate of dope THF1, does not traverse the miscibility gap in a significant way, which is consistent with the observed absence of a bulk porosity (Figure 2e). In contrast, the green and blue curves already at early times reach deep into the unstable region. Hence, for THF3 and THF4 pronounced phase separation is indeed expected. The interpretation of the fate of dope THF2, represented by the yellow curve, is more ambiguous. The trajectory starts in the single phase region but reaches into the miscibility gap. The fact that in spite of this no phase separation is observed for THF2 (Figure 2f), may be explained by a combination of two factors: i) the composition stays near the spinodal, for which reason the driving force for phase separation remains low in comparison to dopes THF3 and THF4, and ii) very fast solvent evaporation, simply does not allow for sufficient time for significant mass transport before gelation sets in.

Another consequence of the fast evaporation of THF is the lower crystallinity in comparison to the fibers obtained from the DMF‐dopes (see Table 1). Since polymer crystallization is a relatively slow process, fast drying likely results in less perfect crystals. In any case, FTIR spectroscopy of the nanofibers, as presented in the Supporting Information, shows the characteristic *β*‐peak at 840 cm^−1^.^[^ ^24^ ^]^ Furthermore, XRD spectra of DMF and THF nanofibers, Figure S3 (Supporting Information), also shows the typical *β*‐phase characteristic peak at 20.4° for all nanofibers. Interestingly, fibers produced from the DMF2 dope, i.e., with a non‐zero water content, exhibit a higher crystallinity that those obtained from DMF1. Furthermore, we note that the THF‐dopes give fibers with a larger mean diameter compared to the ones produced from the DMF solutions. We speculate the fast evaporation of THF is the reason for achieving thicker fibers because fast evaporation prevents stretching and thinning of the electrospinning jet.

### 3.3 Energy Harvesting

To investigate the piezoelectric output of P(VDF‐TrFE) power generators, a custom‐built experimental setup has been used. The nanofiber mats with thickness of 50 µm sandwiched between two Al electrodes are positioned under a small (2 cm × 2 cm) plastic hammer. For a porous piezoelectrics, poling could become challenging as the electric field lines will be concentrated on the low permittivity porous regions. However, post‐fabrication poling of the porous P(VDF‐TrFE) nanofibers is of less concern due to the self‐poled nature of the nanofibers. It has been demonstrated through polarized FTIR spectroscopy that electrospun fibers are self‐poled.^[^ ^18^ ^]^ During the electrospinning, PVDF and P(VDF‐TrFE) nanofiber experience substantial unidirectional tensile stress (parallel to the field) which favors all‐trans chain conformation and enhances formation of the polar *β*‐phase. On their whiplash trajectory to the substrate, the electric field is perpendicular to the fibers, which facilitates transversal polarization along the fiber diameter.^[^ ^18^ ^]^ Self‐poling is further enhanced by the electrospinning from solution because the presence of solvent molecules increases mobility of the polymer chains. Nonetheless, the physical origin of the self‐poling in nanofibers is not yet well understood. Moreover, we have performed poling of the fiber mats after electrospinning, but we have not seen any influence of the poling on the voltage output, which is an indication that the fibers are already poled.

**Figure 4:**
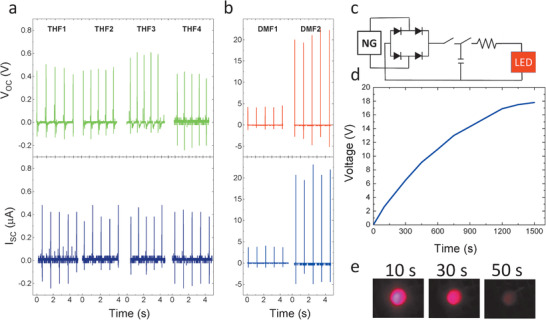
*V*_oc_ and *I*_sc_ of a) THF nanogenerators, b) DMF nanogenerators. All nanofiber layers experienced the same excitation with a mechanical force of 0.2 MPa. The cyclic response of the nanogenerators, *V*_oc_ and *I*_sc_, are given in impact frequency of 1 Hz. c) Schematic circuit diagram of the rectifying bridge used to store the harvested electricity. d) Voltage versus charging time plot for DMF2 nanogenerator connected to a 1 µF capacitor. e) Image of different frames captured from continuous lighting of a LED.

**Figure** **4** presents the open‐circuit voltage (*V*_oc_) and short‐circuit current (*I*_sc_) measured for nanogenerators THF1‐4 and DMF1‐2 under the periodic impact of 0.2 MPa. Irrespective of porosity, THF samples demonstrate *V*_oc_ and *I*_sc_ values below 1 V and 1 µA, respectively. Meanwhile, DMF1 and DMF2 samples show *V*_oc_ and *I*_sc_ values as high as 21 V and 3.8 µA, respectively. To investigate the performance of nanogenerators further, power density values are also calculated. For THF samples power densities are around 5 µW cm^−3^ while DMF1 and DMF2 demonstrate power densities of 0.02 and 5.7 mW cm^−3^ at optimized external load of 1 MΩ, respectively (Figure S4, Supporting Information). Considering the DMF1 sample with solid core and smooth surface as a reference, the power output of nanogenerators composed of fibers with surface porosity, the THF1‐4 series, are significantly reduced, whereas those with inner porosity, DMF4, show a substantial increase. It seems that surface porosity deteriorates the output of nanogenerators. We will illustrate later that for the fibers with surface porosity, at least for the THF1 sample, similar output voltage comparable to DMF1 is expected. Therefore, it can be speculated that the experimentally measured much lower voltages for the THF samples is related to reduced contact area between the nanofibers with surface porosity and the electrode. DMF2 fibers are efficient in harvesting the energy from mechanical vibrations as evidenced in Figure 4b. To demonstrate suitability of DMF2 nanofibers for practical applications, a nanogenerator based on DMF2 sample have been employed to harvest energy from mechanical impact of 0.2 MPa pulses. The generated voltage is rectified and stored in a 1 µF capacitor, Figure 4c. The capacitor plates reach a voltage difference of 19 V, Figure 4d, after only 1500 pulses (with frequency of 1 Hz). The stored energy can light up a commercial LED, Figure 4e, for 50 s (Video S1, Supporting Information). It should be noted that the amount harvested energy that can be consumed depends very much on the harvesting/storing circuitry. Using specifically designed circuits for low frequency mechanical vibrations, even better performances can be expected.^[^ ^26^ ^]^ Finally, we have measured the durability of the nanogenerators based on DMF2 fibers, and observed no change in *V*_OC_ and *I*_SC_ during continuous operation for 5 h, as shown in Figure S5 (Supporting Information). Thanks to the chemical stability of P(VDF‐TrFE), the nanofibers did not show no any degradation when stored at room temperature and relative humidity of 45–50%for a period of three years.

**Figure 5:**
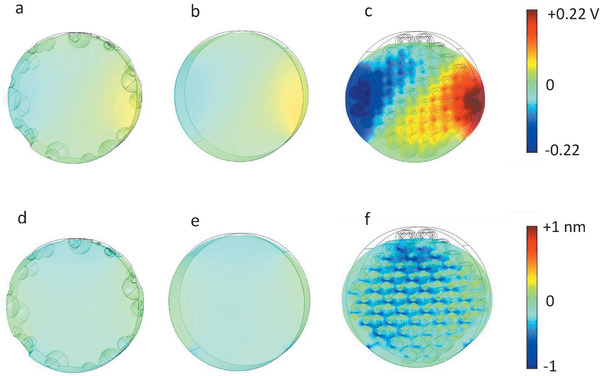
Simulated voltage and volume strain for nanofibers with a,d) solid‐core, b,e), only surface porosity, and c,f) with 45% bulk porosity, respectively. Samples are deformed under the cyclic pressure of 0.2 MPa in the strain‐charge mode. It is assumed that the fibers are self‐poled along the diameter and perpendicular to the nanofiber length.

To understand the influence of hierarchical porosity on the performance of nanogenerators modelling using a finite element method (FEM) is performed. Details of the calculation, the mesh and the parameters are given in the Supporting Information. The complex experimental situation of the nanofiber mat is simplified to a nanofiber with a length of 500 nm. The nanofibers are self‐poled with the polarization direction parallel to the applied mechanical stress. The voltage output and volumetric strain in a section of the nanofiber is calculated for a 0.2 MPa cyclic pressure. The electrical output of real nanofiber mats is generated from a complex network of such sections connected in series and parallel. We have calculated the voltage generation for both strain‐charge and stress‐charge modes. Here, only the result for the stress‐charge form is presented, because it closely resembles the experimental reality. **Figure** **5**a–c compares the generated voltage profile and deformation, Figure 5d–f, of the nanofibers with different porous structures. The solid‐core nanofiber and the nanofiber with surface porosity generate a voltage that is comparable that are of the order of 22 and 27 mV, respectively. The FEM calculation suggests that surface porosity has no effect on the performance of the nanogenerators at low porosity levels. At high porosity levels, ≈50%, the fibers can show similar high voltages. However, due to surface porosity, the contact area between the fibers and the electrodes are drastically reduced. Hence, the experimentally observed significant drop in the output voltage of THF samples is due to the lack of a good contact (due to surface porosity) between the Al electrodes (foils) and the nanofibers. The nanofiber with 45% inner porosity (solid closed shell) generates much higher simulated voltage of 220 mV as shown in Figure 5c (see also Video S2, Supporting Information). It is striking that the calculated increase in the output voltage closely resembles the experimental reality, i.e., the sample with inner porosity of 45%, shows tenfold increase in the output voltage as compared to the one with solid‐core nanofibers. The bulk porosity significantly increases the output voltage of the nanofiber. The enhancement of the power output with increasing porosity is related to the lowered relative permittivity and the enhanced deformability of the nanofibers. For a porous dielectric film consisting of spherical closed pores,^[^ ^17^ ^]^ it is predicted that the relative permittivity, *ε*′, decreases monotonically with the fractional porosity, *P*, as^[^ ^25^ ^]^

(1) $$\varepsilon^{\prime }=\varepsilon \left(1-\frac{3P\left(\varepsilon -1\right)}{P\varepsilon +1-P+2\varepsilon }\right)$$

where, *ε* is the relative dielectric permittivity of P(VDF‐TrFE). For the ideal case of uniformly distributed isolated spherical pores, the dielectric permittivity continuously drops as porosity increases. For instance, for a P(VFD‐TrFE) film, the relative permittivity drops from 10 to 2.7 for a film that is 45% porous. Porous nanogenerators of sample DMF2 have shown 280 times increase in output power in comparison to solid‐core DMF1 nanofiber, for which the reduced effective dielectric constant is partly responsible.

The second contribution is due to the substantial change in stiffness of the porous nanofibers in comparison with a solid core fiber. The piezoelectric charge coefficient *d*_33_, is associated to the ratio of change in applied pressure to change in surface charge *σ*

(2) $$d_{33}=P\left[\frac{\partial ln\mu }{\partial \sigma_{3}}-\frac{\partial lnz}{\partial \sigma_{3}}\right]$$

where *µ*, *z*, and *P* $=\frac{\mu }{\mathrm{volume}}\;$ are total dipole, thickness, and polarization, respectively.^[^ ^18^ ^]^ The first term of Equation (2) is related to the dipole moment of the fiber at constant thickness. The second term is the elastic compressibility constant *σ*_33_, which is related to volumetric strain. Therefore, we have calculated the volumetric strain for the nanofibers under the same 0.2 MPa pressure pulse.

The FEM calculations of the volumetric strain for nanofibers with solid‐core, surface and bulk porosities are presented in Figure 5d–f. The calculation clearly shows large deformation of the nanofiber with 45% bulk porosity whereas for the other two cases only minor deformation is obtained. Substantially increased volumetric strain of the porous nanofibers therefore plays a pivotal rule to increase the power output piezoelectric nanogenerators. Finally, it should be noted that the calculated output voltage using strain‐charge mode instead of stress‐charge mode gives lower voltages (almost a factor of 2) for all nanofibers. The trend observed for both calculations are the same; the voltage increases with increasing the internal porosity due to lowered effective relative permittivity and enhanced volumetric strain in the nanofibers.

## 4 Conclusion

In summary, we have provided a detailed study that exploits the thermodynamics and kinetics of liquid‐liquid phase separation during the electrospinning to achieve nanofibers with hierarchical porosity. Full understanding of solvent/ non‐solvent/PVDF ternary phase diagram is the key ingredient to design the process. A careful choice of the solvent and deliberate addition of water (as nonsolvent) allows for precise positioning of the pores only on the surface or only within the bulk of the nanofibers.

Such level of hierarchical control over structuring is of paramount importance in designing functional devices made of porous nanofibers. As an example, we have fabricated and fully characterized piezoelectric nanogenerators from P(VDF‐TrFE) nanofibers, and shed light on the effect of different type of porous structures on the performance. We have shown that nanofibers with high bulk porosity, besides their reduced effective dielectric constant, can accommodate much large volumetric strains and therefore deliver much higher power output. The application of porous nanofibers however is not limited to nanogenerators. The Knowledge can be applied for the design of polymer membranes for instance for batteries.

## Conflict of Interest

The authors declare no conflict of interest.

## Supporting information

Supporting Information

Supplemental Video 1

Supplemental Video 2

## References

[1] M.‐H. Sun , S.‐Z. Huang , L.‐H. Chen , Y. Li , X.‐Y. Yang , Z.‐Y. Yuan , B.‐L. Su , Chem. Soc. Rev. 2016, 45, 3479. 27255561 10.1039/c6cs00135a

[2] a) J. Zhang , T. Zheng , E. Alarçin , B. Byambaa , X. Guan , J. Ding , Y. S. Zhang , Z. Li , Small 2017, 13, 1770249; 10.1002/smll.201701949 PMC5845855 29094479

[3] a) C. H. Lau , K. Konstas , C. M. Doherty , S. Kanehashi , B. Ozcelik , S. E. Kentish , A. J. Hill , M. R. Hill , Chem. Mater. 2015, 27, 4756;

[4] a) B.‐B. Dong , F.‐H. Wang , M.‐Y. Yang , J.‐L. Yu , L.‐Y. Hao , X. Xu , G. Wang , S. Agathopoulos , J. Membr. Sci. 2019, 579, 111;

[5] M. M. Abolhasani , M. Naebe , K. Shirvanimoghaddam , H. Fashandi , H. Khayyam , M. Joordens , A. Pipertzis , S. Anwar , R. Berger , G. Floudas , J. Michels , K. Asadi , Nano Energy 2019, 62, 594.

[6] Z. Zhang , C. Yao , Y. Yu , Z. Hong , M. Zhi , X. Wang , Adv. Funct. Mater. 2016, 26, 6760. 28603477 10.1002/adfm.201602624 PMC5462116

[7] a) Z.‐L. Xu , F. A. Qusay , J. Membr. Sci. 2004, 233, 101;

[8] H. Fashandi , A. Yegane , M. M. Abolhasani , Fibers Polym. 2015, 16, 326.

[9] G. R. Guillen , Y. Pan , M. Li , E. M. Hoek , Ind. Eng. Chem. Res. 2011, 50, 3798.

[10] X. Chen , Y. Xu , W. Zhang , K. Xu , Q. Ke , X. Jin , C. Huang , Nanoscale 2019, 11, 8185. 30985851 10.1039/c9nr01477b

[11] a) J. Roscow , R. Lewis , J. Taylor , C. Bowen , Acta Mater. 2017, 128, 207;

[12] a) Y. Mao , P. Zhao , G. McConohy , H. Yang , Y. Tong , X. Wang , Adv. Energy Mater. 2014, 4;

[13] a) M. Li , H. J. Wondergem , M.‐J. Spijkman , K. Asadi , I. Katsouras , P. W. Blom , D. M. De Leeuw , Nat. Mater. 2013, 12, 433; 23503012 10.1038/nmat3577

[14] P. Ueberschlag , Sens. Rev. 2001, 21, 118.

[15] a) M. Lou , I. Abdalla , M. Zhu , J. Yu , Z. Li , B. Ding , ACS Appl. Mater. Interfaces 2019, 12, 1597; 31840486 10.1021/acsami.9b19238

[16] a) Y. Calahorra , R. A. Whiter , Q. Jing , V. Narayan , S. Kar‐Narayan , APL Mater. 2016, 4, 116106;

[17] a) T. Zeng , X. Dong , C. Mao , Z. Zhou , H. Yang , J. Eur. Ceram. Soc. 2007, 27, 2025;

[18] D. Mandal , S. Yoon , K. J. Kim , Macromol. Rapid Commun. 2011, 32, 831. 21500300 10.1002/marc.201100040

[19] S. K. Karan , R. Bera , S. Paria , A. K. Das , S. Maiti , A. Maitra , B. B. Khatua , Adv. Energy Mater. 2016, 6, 1601016.

[20] H. S. Dehsari , J. Michels , K. Asadi , J. Mater. Chem. C 2017, 5, 10490.

[21] S. P. Adiga , C. Jin , L. A. Curtiss , N. A. Monteiro‐Riviere , R. J. Narayan , Wiley Interdiscip. Rev.: Nanomed. Nanobiotechnol. 2009, 1, 568. 20049818 10.1002/wnan.50 PMC3684197

[22] a) S. Megelski , J. S. Stephens , D. B. Chase , J. F. Rabolt , Macromolecules 2002, 35, 8456;

[23] https://pubchem.ncbi.nlm.nih.gov/compound/dimethylformamide; https://pubchem.ncbi.nlm.nih.gov/compound/8028 (accessed: December 2019).

[24] a) M. M. Abolhasani , M. Naebe , A. Jalali‐Arani , Q. Guo , PLoS One 2014, 9, 88715; 10.1371/journal.pone.0088715 PMC3925160 24551141

[25] R. Richert , Eur. Phys. J.: Spec. Top. 2010, 189, 37.

[26] X. Wang , P. R. Wilson , R. B. Leite , G. Chen , H. Freitas , K. Asadi , E. C. P. Smits , I. Katsouras , P. R. F. Rocha , Energy Technol. 2020, 10.1002/ente.202000114.

[27] a) R. C. G. Naber , K. Asadi , P. W. M. Blom , D. M. de Leeuw , B. de Boer , Adv. Mater. 2010, 22, 933; 20217816 10.1002/adma.200900759

[28] I. Katsouras , K. Asadi , M. Li , T. B. van Driel , K. S. Kjaer , D. Zhao , T. Lenz , Y. Gu , P. W. M. Blom , D. Damjanovic , M. M. Nielsen , D. M. de Leeuw , Nat. Mater. 2016, 15, 78. 26436342 10.1038/nmat4423

[29] S. K. Ghosh , T. K. Sinha , B. Mahanty , D. Mandal , Energy Technol. 2015, 3, 1190.

