# Supplementary material for: Hierarchically Structured Porous Piezoelectric Polymer Nanofibers for Energy Harvesting
Source: Adv Sci (Weinh). 2020 Jun 3;7(13):2000517. doi: 10.1002/advs.202000517 (PMC7341085; doi:10.1002/advs.202000517)
Supplement: Supplementary file 1 — Supporting Information [file ADVS-7-2000517-s001.pdf]

Supplementary Information

Hierarchically structured porous piezoelectric polymer nanofibers for energy harvesting

Mohammad Mahdi Abolhasani[1, 2*], *Minoo Naebe*[3], *Morteza Hassanpour Amiri*[1], *Kamyar Shirvanimoghaddam*[3], *Saleem Anwar*[1,4], *Jasper Michles*[1], *Kamal Asadi*[1*]

[1]Max-Planck Institute for Polymer Research, Ackermannweg 10, 55128, Mainz, Germany

[2]Chemical Engineering Department, University of Kashan, 8731753153, Kashan, Iran

[3]Carbon Nexus, Institute for Frontier Materials, Deakin University, 3217, Geelong, Australia

[4] School of Chemical & Materials Engineering, National University of Sciences & Technology, Sector H-12, Islamabad, Pakistan

Corresponding Authors

abolhasani@mpip-mainz.mpg.de

asadi@mpip-mainz.mpg.de

S1) Phase diagram of water/DMF/P(VDF-TrFE) and water/THF/P(VDF-TrFE) ternary systems

Calculation of the ternary phase diagrams is conducted based on the Flory-Huggins (FH) Gibbs free energy of mixing (ΔG_M), expanded by Tompa [1] for nonsolvent/solvent/polymer ternary mixtures (Equation S1).

$$\frac{\Delta G_M}{RT} = n_1 \ln\varphi_1 + n_2 \ln\varphi_2 + n_3 \ln\varphi_3 + n_1\varphi_2 g_{12}(u_2) + n_2\varphi_3\chi_{23} + n_1\varphi_3\chi_{13} \tag{S1}$$

where n_i and φ_i represent the number of moles and the volume fraction of component i, respectively; R and T denote to the universal gas constant (8.314 J/mol·K) and the absolute temperature (K), respectively. χ_{23} is the solvent(2)/polymer(3) binary interaction parameter which is considered to be concentration-independent in this work. χ_{13} stands for the nonsolvent(1)/polymer(3) interaction parameter, often assumed as a constant. Finally, $g_{12}(u_2)$ is a generalized nonsolvent(1)/solvent(2) interaction parameter that depends on the solvent composition $u_2 = \varphi_2/(\varphi_1 + \varphi_2)$ of a pseudo binary mixture as follows [2]:

$$g_{12}(u_2) = \frac{\alpha}{\beta - \gamma u_2} \tag{S2}$$

In the present contribution, the $g_{12}(u_2)$, χ_{23} and χ_{13} interaction parameters at 20 ºC are collected from literature [2, 3, 4] and are listed in Table S1. The procedure to calculate the binodal and spinodal curves have adopted from Altena et al. [5] method. The theoretically calculated binodal curve is further verified using the experimentally measured cloud points.

Table S1 *Binary interaction parameters used to construct water/DMF/P(VDF-TrFE) ternary phase diagram at temperature of 20 ºC.*

Ternary mixture	nonsolvent(1)/solvent(2)	χ_{23}	χ_{13}
water/DMF/P(VDF-TrFE)	$g_{12}(u_2) = \frac{0.322}{0.075 - 0.960u_2}$	0.1	2.89
water/THF/P(VDF-TrFE)	$g_{12}(u_2) = \frac{0.590}{0.517 - 0.778u_2}$	0.38	2.89

S2) Calculation of mean composition trajectories

The trajectories of mean composition are calculated using an extension of the pressure deficit model we published earlier[4]. This model assumes the solution to be in equilibrium with the composition of the vapor phase just above it. Nevertheless, the model does not treat the vapor phase explicitly. The magnitude of the ambient partial pressure, p_i^{∞} determines whether mass is lost via evaporation or gained via condensation from the surrounding vapor phase. The model is isothermal and does hence not consider evaporative cooling. In the present work we assume the change in volume fraction of a component i in the mixture to be approximately given by [6]:

$$\frac{\partial \phi_i}{\partial t} = -h^{-1}\left[k_i \frac{\bar{v}_i^{(l)}}{\bar{v}_i^{(g)}} P^{-1}\left(p_i^{\circ}\exp\left(\frac{\Delta\mu_i}{RT}\right) - p_i^{\infty}\right) + \phi_i \frac{\mathrm{d}h}{\mathrm{d}t}\right] \tag{S3}$$

Here, p_i° and P are the vapor pressure of the pure component i and P the ambient pressure. $\bar{v}_i^{(l)}$ and $\bar{v}_i^{(g)}$, *i.e.* the partial specific volume of component i in the liquid and vapor phase, are approximated to be: $\bar{v}_i^{(l)} \approx 1$ cm^3/g (for THF, DMF and water), and $\bar{v}_i^{(g)} \approx V_{\mathrm{m}}^{(g)}/M_i$ (with M_i the molar mass and $V_{\mathrm{m}}^{(g)}$ the ideal gas molar volume, which at 20 $^{\circ}$C: $V_{\mathrm{m}}^{(g)}$ = 0.023 m^3/mol), which

amounts to ~3 and ~12 cm^3/g for both solvents and water, respectively. The length h represents the time-dependent cross sectional dimension of the specimen from/into which liquid/vapor evaporates/condenses. In all calculations we use $h(t = 0)$ = 10 μm and note that its value only affects the absolute time scale of evaporation and not the curvature or directionality of the trajectory of mean composition. As proposed by Tsay and McHugh [6], the mass transfer coefficient k_i is a function of the viscosity of the vapor phase (which time-dependence we neglect), the vapor phase diffusivity, as well as the geometry of the specimen or film. Since here we are only interested in relative comparison and not in absolute time scales, we treat k_i as effective parameters.

The values of the pressure parameters and mass transfer coefficient ratios used in the calculations are listed in Table S2. We assume the ambient partial pressures of THF and DMF to be zero, *i.e.* implying that evaporation is not limited by vapor diffusion. A solvent-to-water mass transfer ratio of ~0.5 is a rough intuitive estimate, which assumes $k_{\mathrm{solvent}}/k_{\mathrm{H_2O}}$ to scale with the ratio of the vapor phase diffusivities of water and solvent raised to a power of order unity [6]. As mentioned in the main document, we consider the possibility that for the current system these assumptions may not be valid, upon which we attempt to capture deviations by adjusting the mass transfer coefficients: the alternative value of $k_{\mathrm{solvent}}/k_{\mathrm{H_2O}} \approx 5$ is a "fitting" value in order to computationally discriminate between the trajectories associated with the two DMF dopes (dashed lines in Figure 1b in the main document). A similar situation holds for the ambient partial water pressure in case of the DMF dopes: the value of 1.2 kPa corresponds to "typical" laboratory conditions of 20 ^{o}C/50% RH. The five times lower effective value of 0.23 kPa is used to calculated the dashed lines in Figure 1b (the main document).

Table S2 *Input parameters for pressures and mass transfer coefficient ratios*

Parameter	Value
P	101.33 kPa
$p^{\circ}_{\mathrm{THF}}(20\ °\mathrm{C})$	20 kPa [7]
$p^{\circ}_{\mathrm{DMF}}(20\ °\mathrm{C})$	0.5 kPa [8]
$p^{\circ}_{\mathrm{H_2O}}(20\ °\mathrm{C})$	2.3 kPa [9]
$p^{\infty}_{\mathrm{THF}}$	0 Pa
$p^{\infty}_{\mathrm{DMF}}$	0 Pa
$p^{\infty}_{\mathrm{H_2O}}$	1.2 kPa / 0.23 kPa
$k_{\mathrm{THF}}/k_{\mathrm{H_2O}}$	0.5
$k_{\mathrm{DMF}}/k_{\mathrm{H_2O}}$	0.5 / 5

The chemical potentials $\Delta\mu_i$ (Equation (3)) are calculated as described in ref. [10] using Flory-Huggins theory, assuming normalized molecular sizes of $N_{\mathrm{H_2O}}$ = 1, N_{THF} = N_{DMF} = 4 and N_{polymer} = 1000 and interaction parameters are treated constant and are given in Table S3.

Table S3 *Flory-Huggins interaction parameters used for calculating the trajectories of mean composition*

	THF	DMF	H_2O	P(VDF-TrFE)
THF	0	-	0.5	0.4
DMF	-	0	0.5	0.1
H_2O	0.5	0.5	0	2.8
P(VDF-TrFE)	0.4	0.1	2.8	0

S3) Thermoporometry technique

Total pore volume of porous fibers are calculated using Eq. 1 in which ΔH_{pore}, ΔH_{excess}, m_{liq}, m_{solid}, ρ_{liq} denote to the pore melt enthalpy, excess melt enthalpy, probe liquid mass, fiber dry mass and probe liquid density, respectively [11].

$$V_P = \frac{\Delta H_{Pore}}{\Delta H_{Pore}+\Delta H_{excess}} \frac{m_{liq}}{m_{solid}} \frac{1}{\rho_{liq}} \tag{S4}$$

Using m_{solid} and density of polymer ($\rho_{P(VDF\text{-}TrFE)}$=1.78 g/cm^3), the volume fraction of pores within different porous fibers are evaluated and presented in Table 1.

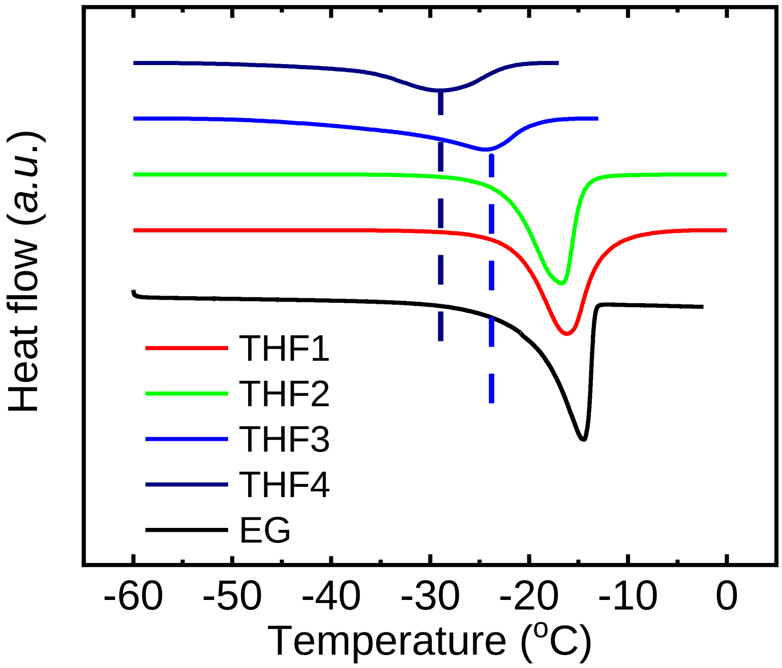

Fig. S1. *Thermoporometry graph of EG and THF samples. Dash lines present melting point depression of EG inside the THF3 and THF 4 pores. THF1 and THF2 behavior are similar to EG demonstrate non-porous structure of these fibers.*

S4) FTIR spectroscopy

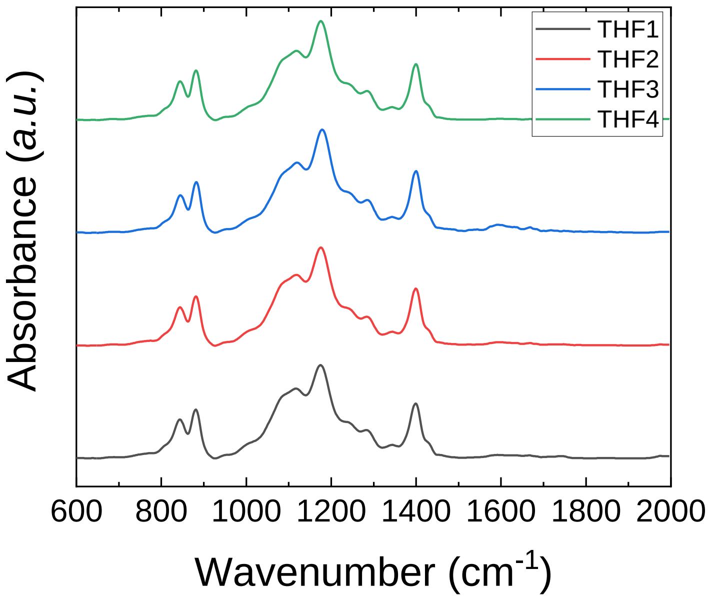

Fig. S2. *FTIR spectrum of P(VDF-TrFE) nanofiber mats prepared using THF.*

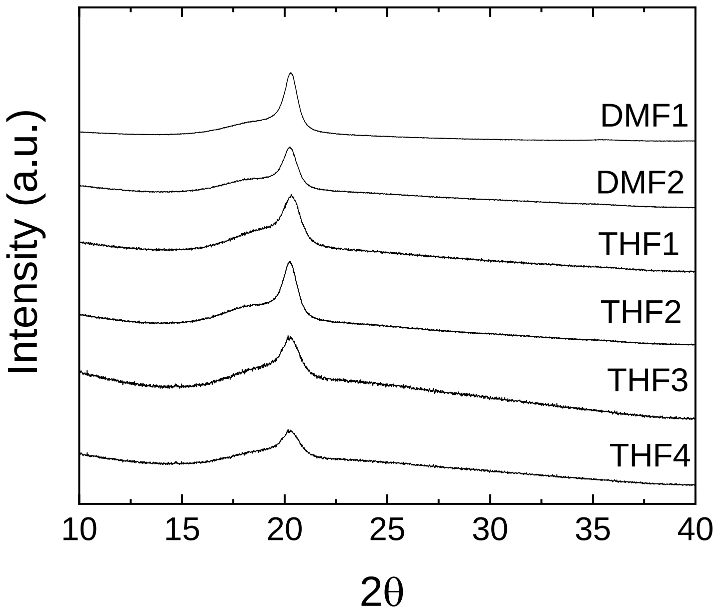

Fig. S3. *XRD diffractograms of the DMF and THF samples.*

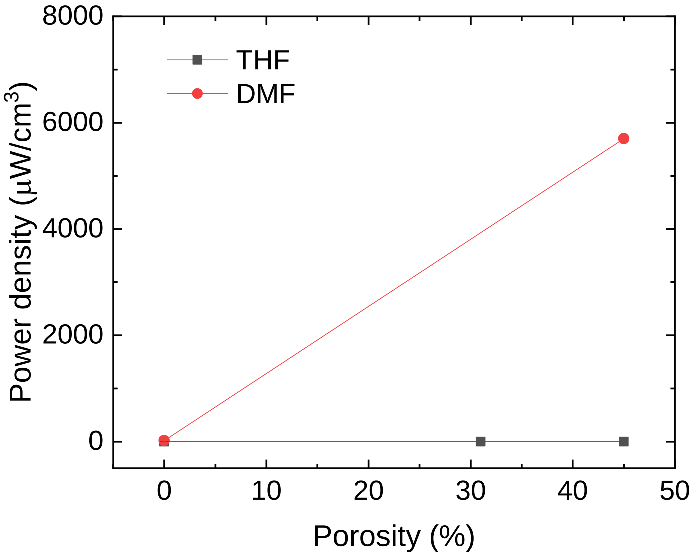

Fig. S4 *Power density vs. Porosity (%)for DMF and THF samples. Power densities calculated at optimum resistance of 1MΩ*

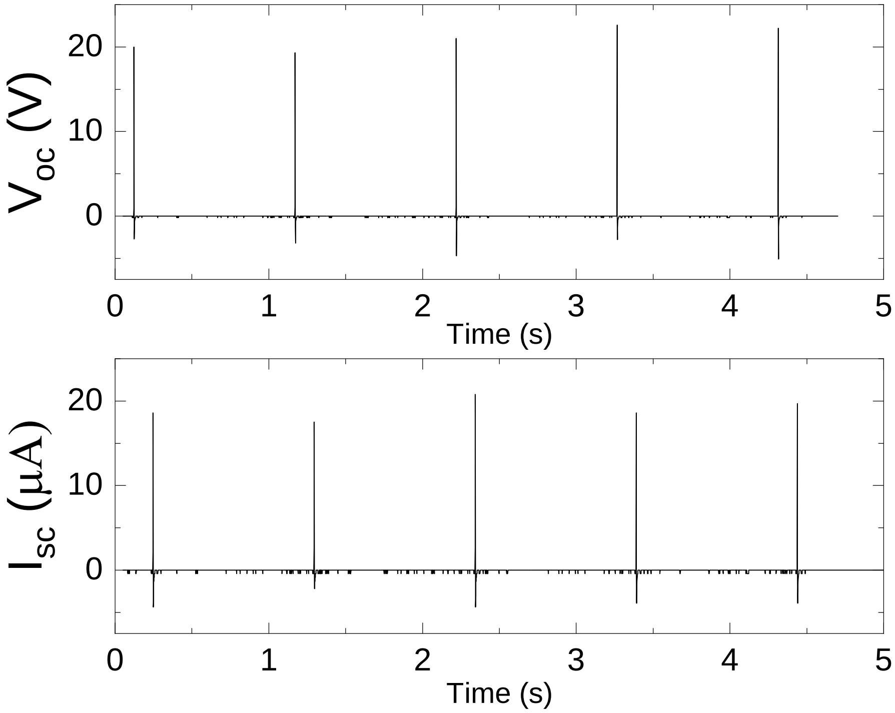

Fig. S5. *Open circuit voltage and short circuit current of DMF2 sample after impacting the nanogenerator for five hours at mechanical impact of 0.2 MPa.*

S5) The simulation detail have conducted using the COMSOL Multiphysics software package

To simulate the volumetric strain and piezoelectric potential of nanofibers a unit of nanofibers with 500nm length, with different porosities equal to DMF1, THF1, DMF2 samples are modeled as presented in Figure S6 Required parameter for simulation have extracted from literature [12] and have presented in Table S4.

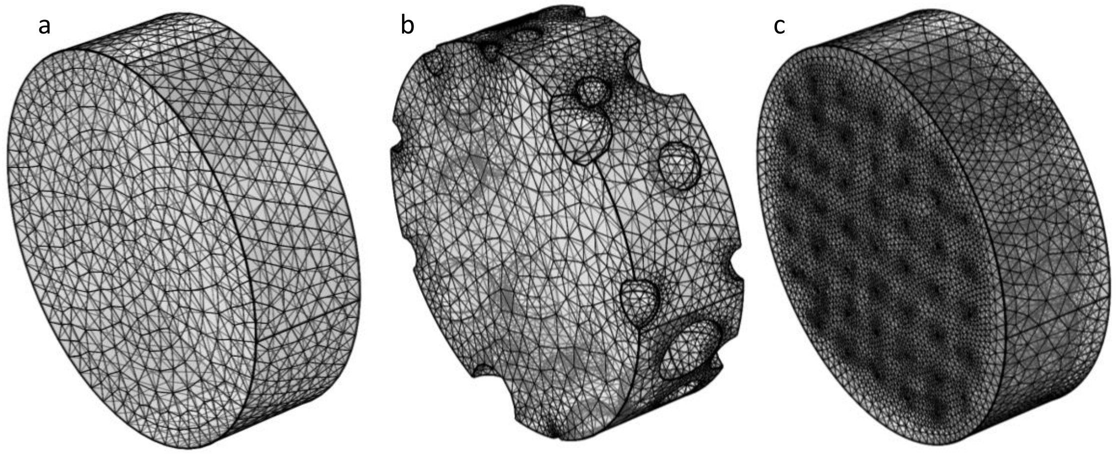

Fig. S6. *unit models of a) sample without porosity (DMF1), b) sample with surface porosity (THF1), c) sample with 45% porosity (DMF2), after meshing.*

Table S4. P(VDF-TrFE) parameters from COMSOL library have been used for simulation

Property	Variable	Expression	Unit
Elasticity matrix	C_E	$\begin{matrix} 3.61e9 & 1.61e9 & 1.42e9 & 0 & 0 & 0 \\ 1.61e9 & 3.13e9 & 1.31e9 & 0 & 0 & 0 \\ 1.42e9 & 1.31e9 & 1.63e9 & 0 & 0 & 0 \\ 0 & 0 & 0 & 0.55e9 & 0 & 0 \\ 0 & 0 & 0 & 0 & 0.59e9 & 0 \\ 0 & 0 & 0 & 0 & 0 & 0.69e9 \end{matrix}$	Pa
Coupling matrix	e	$\begin{matrix} 0 & 0 & 0 & 0 & -0.0113 & 0 \\ 0 & 0 & 0 & 0.0110 & 0 & 0 \\ 0.0098 & -0.0109 & -0.0263 & 0 & 0 & 0 \end{matrix}$	C/m^2
Density	ρ	1780	kg/m^3
Relative permittivity	ε_r	10 for neat P(VDF-TrFE)	1

The lower electrode is fixed and the bottom of the P(VDF-TrFE) fiber was electrically grounded. An equal external pressure of 0.2 MPa is applied to the top of unit of the nanofibers with 500nm length. Using COMSOL Multiphysics the piezoelectric, electric and elastic equations are solved to obtain the volumetric strain and piezoelectric potential.

References

[1] H. Tompa, Transactions of the Faraday Society 1949, 45, 1142.

[2] M. Karimi, W. Albrecht, M. Heuchel, M. Kish, J. Frahn, T. Weigel, D. Hofmann, H. Modarress, A. Lendlein, Journal of membrane science 2005, 265, 1.

[3] 조재환, Polymer (Korea) 1991, 15, 67; M. M. Abolhasani, M. Naebe, K. Shirvanimoghaddam, H. Fashandi, H. Khayyam, M. Joordens, A. Pipertzis, S. Anwar, R. Berger, G. Floudas, J. Michels, K. Asadi, Nano Energy 2019, 62, 594.

[4] H. S. Dehsari, J. J. Michels, K. Asadi, Journal of Materials Chemistry C 2017, 5, 10490.

[5] F. W. Altena, C. Smolders, Macromolecules 1982, 15, 1491.

[6] C. Tsay, A. McHugh, Journal of membrane science 1991, 64, 81.

[7] https://pubchem.ncbi.nlm.nih.gov/compound/8028#section=Vapor-Pressure.

[8] https://pubchem.ncbi.nlm.nih.gov/compound/dimethylformamide#section=Vapor-Pressure.

[9] https://www.chegg.com/homework-help/questions-and-answers/collect-following-data-complete-experiment-water-temperature-230-c-gas-volume-cylinder-rea-q15828045.
[10] C. Schaefer, J. J. Michels, P. van der Schoot, Macromolecules 2016, 49, 6858.
[11] M. Iza, S. Woerly, C. Danumah, S. Kaliaguine, M. Bousmina, Polymer 2000, 41, 5885.
[12] X. Chen, H. Tian, X. Li, J. Shao, Y. Ding, N. An, Y. Zhou, Nanoscale 2015, 7, 11536.
